# NUDCD1 knockdown inhibits the proliferation, migration, and invasion of pancreatic cancer via the EMT process

**DOI:** 10.18632/aging.203276

**Published:** 2021-07-29

**Authors:** Chunling Shi, Min Weng, Hengyue Zhu, Yangyang Guo, Dongdong Xu, Hairu Jin, Binshuang Wei, Zhensheng Cao

**Affiliations:** 1 School of Stomatology, Wenzhou Medical University, Wenzhou 110013, China; 2 Key Laboratory of Diagnosis and Treatment of Severe Hepato-Pancreatic Diseases of Zhejiang Province, The First Affiliated Hospital of Wenzhou Medical University, Wenzhou 325000, China

**Keywords:** pancreatic cancer, NUDCD1, EMT, apoptosis, proliferation

## Abstract

NudC domain containing 1 (NUDCD1) is an oncoprotein frequently activated or upregulated in various human cancers, but its role in pancreatic cancer (PC) remains unknown. Thus, we aimed to determine the function and mechanism of NUDCD1 in PC. We employed Western blot and quantitative real-time polymerase chain reaction to assess NUDCD1 expression in cells and PC tissues. NUDCD1 was knocked down in Patu8988 and PANC-1 cells. We conducted real-time cell analysis, wound healing assay, transwell assay and colony formation assay to evaluate the metastatic and proliferative abilities of PC cells. Western blot was conducted to assess the expression of markers associated with apoptosis and epithelial–mesenchymal transition (EMT). Also, we established a tumor xenograft model to determine the role of NUDCD1 *in vivo*. NUDCD1 was overexpressed in PC tissues and cells. NUDCD1 knockdown suppressed the invasion, migration, and proliferative abilities of the cells and induced PC cell apoptosis. The specific mechanism of NUDCD1 was related to the modulation of the EMT process. Data obtained from *in vivo* experiments revealed that NUDCD1 knockdown inhibited the tumor growth, proliferation, and metastasis by modulating the EMT and inducing the apoptosis of PC cells.

## INTRODUCTION

Pancreatic cancer (PC) is a cancer that is highly resistant to chemotherapy, grows rapidly, and undergoes early distant metastasis [1, 2]. Patients with PC have extremely poor prognosis because of distant metastasis and the high frequency of tumor recurrence [3, 4]. Although multiple genes in tumor recurrence and metastasis have been identified, the underlying mechanism of PC metastasis remains unclear. Looking for reliable biomarkers for recurrence and metastasis will greatly benefit patients with PC and may provide novel gene targets for PC treatment.

NudC domain containing 1 (NUDCD1, also known as CML66 or OVA66) is an oncoprotein that is frequently activated or upregulated in various human cancers and well known as an important cancer antigen [5–7]. NUDCD1 is involved in the regulation of different biological processes, such as apoptosis and immune responses [8]. NUDCD1 overexpression enhances the proliferation, invasion, and survival of HO-8910 cells [9]. Although current evidence suggests that NUDCD1 may act as an oncoprotein, its expression and molecular mechanisms in PC metastasis remain unclear.

We hypothesized that NUDCD1 plays a crucial function during the progression of PC. We measured NUDCD1 expression in human PC tissues and determined its contribution to PC proliferation, invasion, and migration *in vitro* and *in vivo* to test our hypothesis. Our data revealed that NUDCD1 is upregulated in PC cells and tissues and plays a major metastatic role in growth, epithelial–mesenchymal transition (EMT), and PC cell apoptosis.

## MATERIALS AND METHODS

### Cell culture and experimental reagents

Human PC cell lines PANC-1, CFPAC-1, and Patu8988 (ATCC, Manassas, VA, USA) were grown in Dulbecco’s modified Eagle medium (DMEM; Invitrogen, Carlsbad, CA) that contained 10% fetal bovine serum and 1% penicillin–streptomycin (Sigma, Taufkirchen, Germany). Primary antibodies for NUDCD1 (Ab126902, 1:1000) and GAPDH (Ab8245, 1:2000) were obtained from Abcam. Antibodies against human Bcl-2 (12789-1-AP, 1:1000), Bax (50599-2-Ig, 1:1000) and Caspase 3 (19677-1-AP, 1:1000) were purchased from Proteintech. Antibodies against vimentin (AF7013, 1:1000), N-cadherin (AF4039, 1:1000), Cleaved-Caspase 3 (AF7022, 1:1000) and E-cadherin (AF0131, 1:1000) were purchased from Affinity Biosciences.

### SiRNA transfection

The cell lines PANC-1 and Patu8988, which highly express NUDCD1, were screened from various pancreatic cell lines by Western blot and quantitative real-time polymerase chain reaction (qRT-PCR). SiRNAs that target NUDCD1 were synthesized by GenePharma (Shanghai, China). The primer sequences of the siRNAs were as follows: NUDCD1 siRNA1, 5′-GGUGCUAGGUAGAAAGUUAGG-3′ (sense) and 5′-UAACUUUCUACCUAGCACCUC-3′ (antisense); NUDCD1 siRNA2, 5′-GAUAGUACUAGCAAGUAUACU-3′ (sense) and 5′-UAUACUUGCUAGUACUAUCUU-3′ (antisense); NUDCD1 siRNA3, 5′-AGAUAUGUAUCUUAUAUAAAC-3′ (sense) and 5′-UUAUAUAAGAUACAUAUCUUG-3′ (antisense). We transfected the PANC-1 and Patu8988 cells with siRNAs using Lipofectamine 3000 (Life Technologies) as per the instructions of the manufacturer for 48h. The efficiency of siRNA transfection was assessed by qRT-PCR.

### Immunohistochemistry (IHC) assay

We conducted IHC according to the methods described previously [10]. In brief, sections were fixed in formalin, embedded in paraffin, and stained with antibody against NUDCD1 (1:100). The primary antibody was substituted with preimmune IgG serum in negative controls.

### Real-time cell analysis (RTCA)

The cell proliferation of the NUDCD1 knocked down Patu8988 and PANC-1 cells was measured by a commercially available RTCA kit as per the manufacturer’s protocol. We first determined the background level by loading 200 μL/well of culture medium (DMEM with 10% FBS) into a 16-well E-plate. Cells (about 10,000 cells/well) were seeded in the E-plate and cultured for 24 h. Cell index was measured every 15 min. Changes in the number and activity of cells were reflected by the detection of the cellular index of reactive cell activity.

### Colony formation assay

We seeded PC cell lines (with or without siRNA transfection) into 6-well plates (600 cells/well). Subsequently, we cultured the cells at 37° C in complete medium in a humidified atmosphere with 5% CO_2_. The medium was refreshed at a 2-day interval. The culture was maintained for 2 weeks, and the colonies were stained with crystal violet solution before the number of clones was counted. This data was then used to determine the cell proliferation rate.

### Flow cytometry analysis

An apoptosis detection kit (Pharmingen, San Diego, CA, USA) was used to conduct propidium iodide (PI) and annexin V–fluorescein isothiocyanate (FITC) staining to determine the rate of cell apoptosis. Subsequently, flow cytometric analysis was performed following NUDCD1 knockdown in PANC-1 and Patu8988 cells as per the manufacturer’s protocol. In brief, the cells (5×10^5^ cells/well) were grown for 24 h and then stained with Annexin V. Next, the cells were rinsed with PBS, then resuspended in a binding buffer (100 mL) with 5 μL FITC and 5 μL PI, and incubated for 30 min in the dark. Finally, a FACScalibur flow cytometer was used to analyze the cells after adding 400 μL of binding buffer.

### Wound healing assay

Wound healing assay was conducted to evaluate the migration ability of the tumor cells. After transfection, the cells were seeded into 6-well plates and incubated for 48 h. A sterile pipette tip (100 μL) was used to scratch the surface of a monolayer to create wounds. Debris and keratinocytes were washed off with PBS, and the medium was substituted with serum-free DMEM. Finally, we examined the wound area and took photographs at 0 and 24 h to determine the number of migrated cells.

### Transwell invasion assay

Transwell invasion assay was used to assess the invasive capacity of the cells. In brief, the PANC-1 and Patu8988 cells with serum-free DMEM medium were seeded into the upper chamber after NUDCD1 knockdown. Matrigel (1:8 diluent of 50 mg/L) was coated on the upper surface of the bottom membrane of the Transwell chamber and dried at 4° C. We filled the bottom wells with complete DMEM. The cells in the upper chamber were removed after 24 h of incubation. The cells that moved via the Matrigel matrix membrane were fixed in 4% paraformaldehyde and stained with 0.1% crystal violet. Finally, the cells were visualized under a microscope and photographed. We randomly selected five fields and counted the number of cells in each field.

### Gene expression analysis

TRIzol (Invitrogen) was used to extract total RNA from the knocked down cells and control cells. The RNA was further purified using the RNeasy Mini Kit and RNase-free DNase Set (Qiagen, Valencia, CA, USA) as per the instructions of the manufacturer. Next, the RNA was reverse-transcribed to cDNA using a cDNA synthesis kit (Promega M-MLV cDNA) as per the protocol of the manufacturer. Subsequently, we conducted qRT-PCR assay to assess NUDCD1 mRNA expression. β-actin served as the internal control for reference. The following are the sequences of the primers used: NUDCD1, 5′-CAGCCCTTTGTGAGTGCCTTCG-3′ (forward) and 5′-GTCCTACTTGCCTGCCTTCCTTTC-3′ (reverse); β-actin, 5′-TGACGTGGACATCCGCAAAG-3′ (forward) and 5′-CTGGAAGGTGGACAGCGAGG-3′ (reverse).

### Western blot analysis

We performed Western blot according to the methods described by Nina Kočevar et al [11]. The cells were removed from the culture plates, resuspended in PBS, and collected via centrifugation. Cell lysis was done using lysis buffer at 4° C for 20 min. We sonicated and extracted the cells via 30-min high-speed centrifugation at 4° C to remove cell debris. Protein assay system (Bio-Rad, Hercules, CA, USA) was employed to evaluate the protein concentration. Subsequently, the proteins (50 mg) were resolved by sodium dodecyl sulfate–polyacrylamide gel electrophoresis and transferred onto nitrocellulose membranes. The membranes were incubated with antibodies specific for the desired proteins. The proteins were detected using an enhanced chemiluminescence Western blot kit (Pierce, Rockford, IL, USA) as per the manufacturer’s protocol.

### Tumor formation in nude mice

Twelve BALB/CA nude mice (weight, 16–20 g; age, 6–8 weeks) were randomized into three groups: Si-NC, Si-RNA1, and Si-RNA2. We injected the cell suspension (100 μL, 5×10^6^ cells) subcutaneously into the right axilla of each mice. Tumorigenesis in the mice was assessed weekly. The mice were sacrificed via CO2 asphyxiation 4 weeks post-injection, and the tumors were extracted to determine their weight and volume (volume = 1/2×length×width^2^).

### Data analysis

Data were analyzed using GraphPad software. Student’s t-test was used to assess the difference between the experimental and control groups. Statistical significance was set at p < 0.05.

## RESULTS

### Expression and clinical importance of NUDCD1 in pancreatic ductal adenocarcinoma (PDAC)

**Figure 1:**
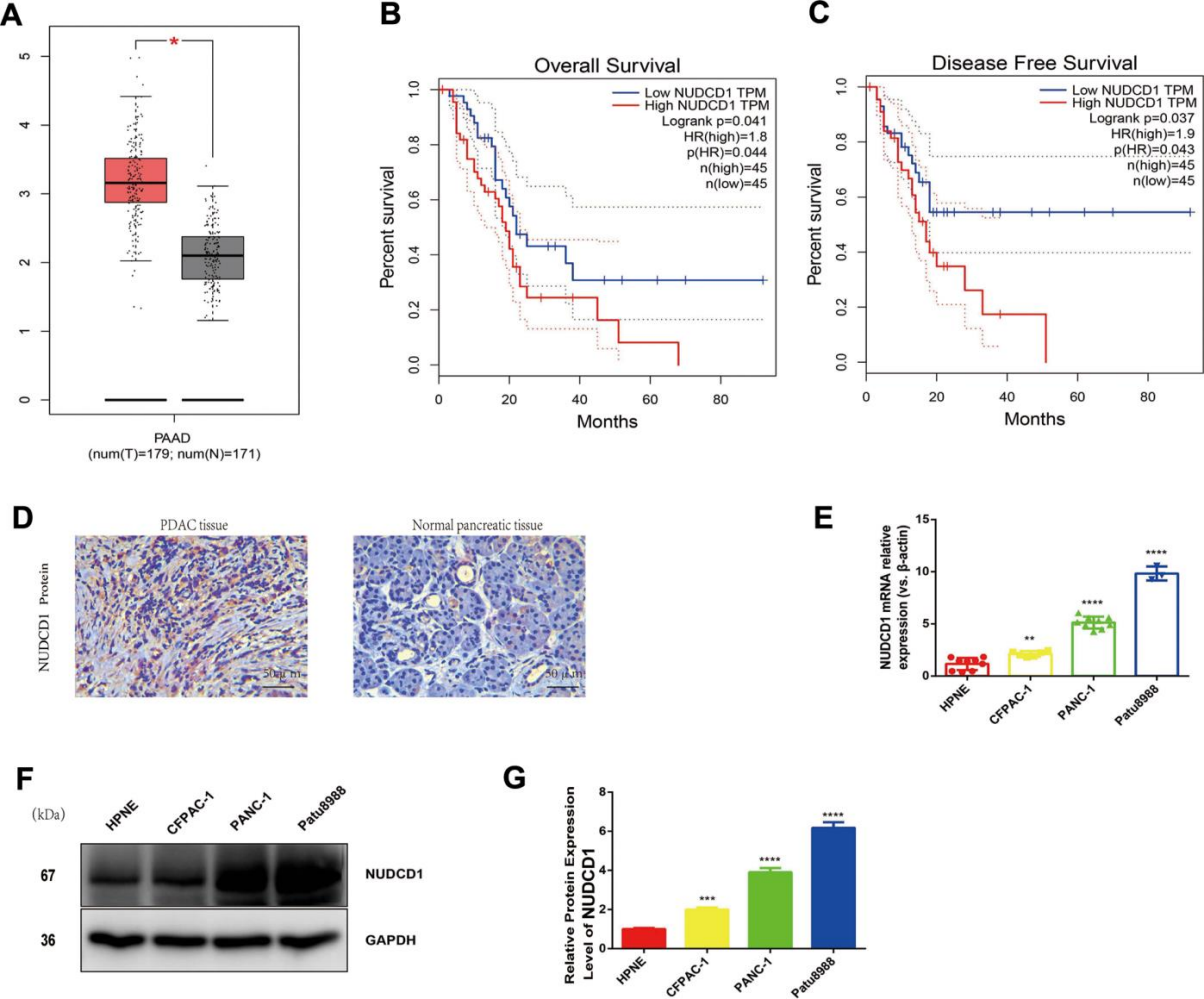
**Expression and roles of NUDCD1 in PC.** (**A**) NUDCD1 mRNA expression levels were significantly higher in PDAC tissues (n = 179) than in normal pancreatic tissues (n = 171). (**B**, **C**) NUDCD1 upregulation was significantly correlated with shorter overall survival and shorter disease-free survival (p = 0.041 and 0.037, respectively). (**D**) NUDCD1 was overexpressed in PDAC tissues relative to adjacent tissues as detected by IHC. (**E**) NUDCD1 upregulation was observed in different PC cell lines by qRT-PCR. (**F**, **G**) High NUDCD1 expression was observed in different PC cell lines by Western blot. *p<0.05, **p<0.01, ***p<0.001, ****p<0.0001.

Herein, we employed the Gene Expression Profiling Interactive Analysis server (http://gepia.cancer-pku.cn/) to mine and analyze NUDCD1 expression data according to the Genotype-Tissue Expression and The Cancer Genome Atlas databases. Based on the analysis, the levels of NUDCD1 mRNA were overtly higher in PDAC tissues in comparison to normal pancreatic tissues (Figure 1A). According to log-rank test, the upregulation of NUDCD1 was significantly related to overall survival (p = 0.041) and disease-free survival (p = 0.037; Figure 1B, 1C). Furthermore, the IHC results revealed that NUDCD1 was overexpressed in PDAC tissues relative to matched adjacent tissues (Figure 1D). We determined the NUDCD1 expression in many PC cell lines versus normal pancreatic cell line HPNE via qRT-qPCR and Western blot. Based on the results, NUDCD1 was markedly upregulated in PANC-1, CFPAC-1, and Patu8988 cells (Figure 1E, 1F), especially in Patu8988 and PANC-1 cells. Collectively, these findings indicated that NUDCD1 is aberrantly overexpressed in PDAC.

### NUDCD1 knockdown inhibits the proliferation and colony formation of PC cells

**Figure 2:**
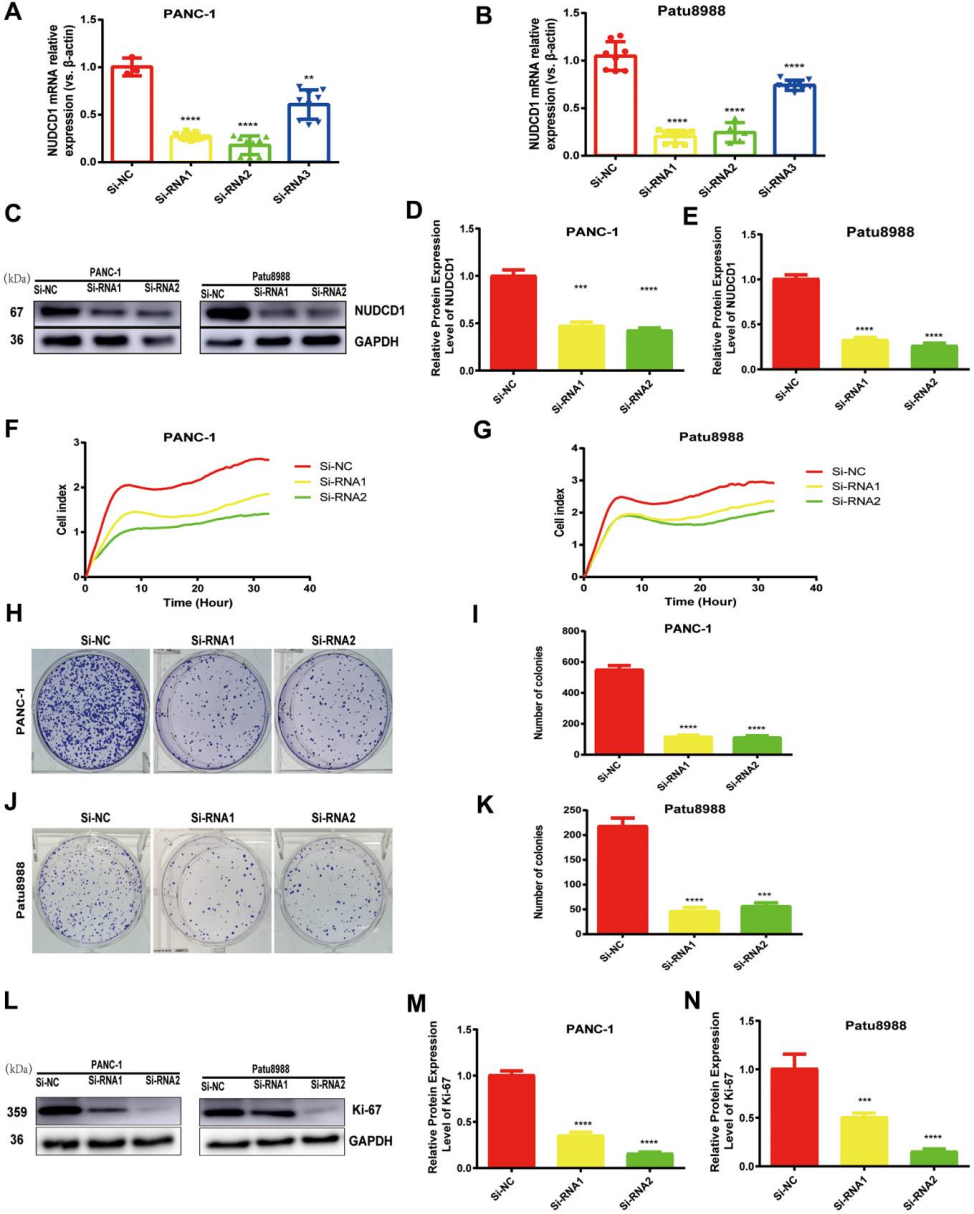
**NUDCD1 knockdown inhibited the proliferation of PC cells.** (**A**, **B**) The interference effects of three siRNAs on NUDCD1 were confirmed by qRT-PCR. (**C**–**G**) NUDCD1 knockdown inhibited the proliferation of PANC-1 and Patu8988 cells. (**H**–**K**) NUDCD1 knockdown inhibited the colony formation of Patu8988 and PANC-1 cells. (**L**–**N**) Western blot analysis showed that NUDCD1 knockdown decreased the expression of Ki-67 in PANC-1 and Patu8988 cells.**p<0.01, ***p<0.001, ****p<0.0001.

Considering that NUDCD1 is usually upregulated in PC, we speculated that NUDCD1 could facilitate PC progression. PANC-1 and Patu8988 cell lines, which highly express NUDCD1, were used to examine the role of NUDCD1 in the pathogenesis of PC. Thus, siRNA1, siRNA2, and siRNA3 were used to knock down NUDCD1 expression. Obviously, siRNA1 and siRNA2 reduced NUDCD1 expression by half or more (Figure 2A, 2B). The RTCA results showed that the silencing of NUDCD1 remarkably inhibited the proliferation of PANC-1 and Patu8988 cells (Figure 2C, 2D). In addition, NUDCD1 gene knockdown obviously reduced the colony formation of PC cells (Figure 2E–2H).

### NUDCD1 gene silencing triggers PC cell apoptosis *in vitro*

**Figure 3:**
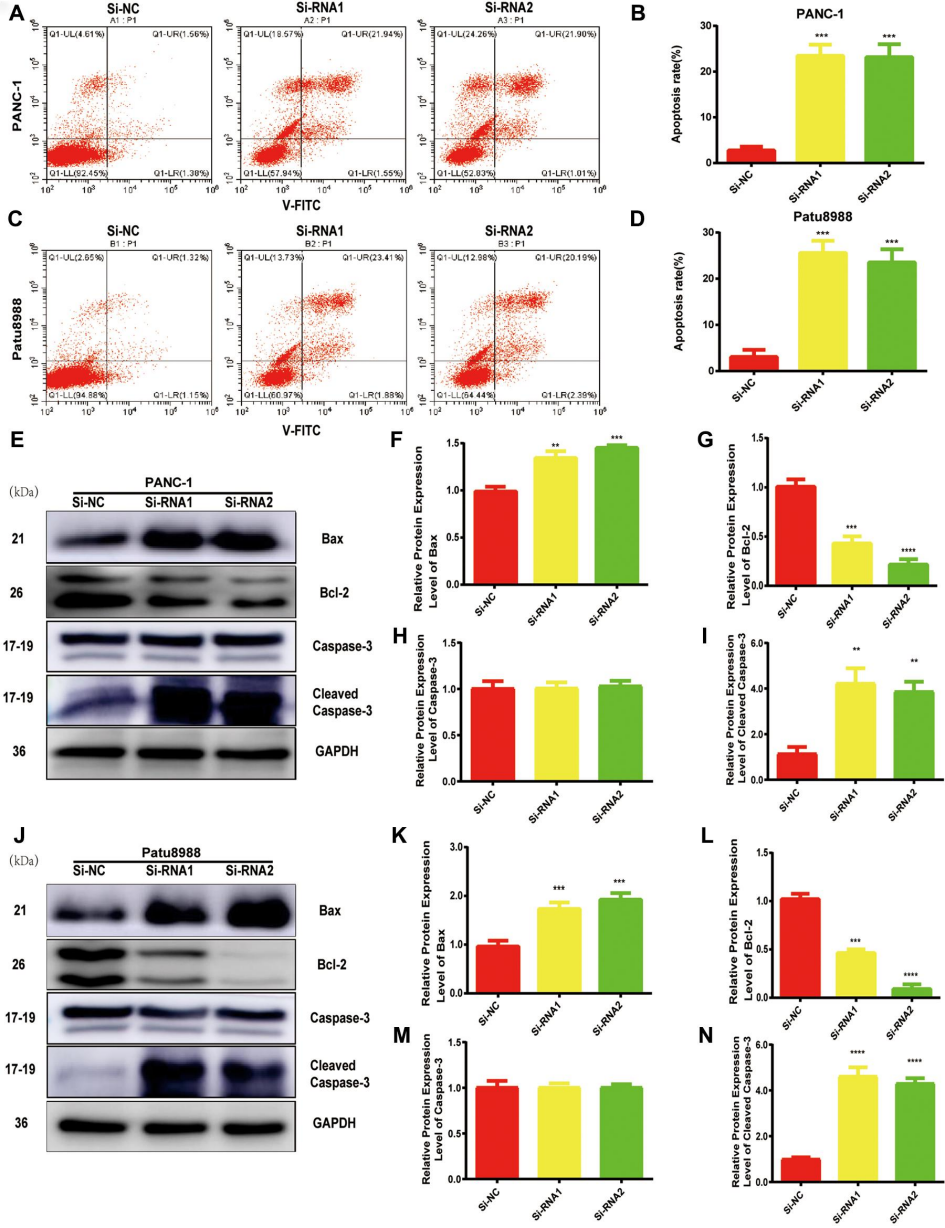
**NUDCD1 knockdown induced the apoptosis of PC cells.** (**A**–**D**) Flow cytometric analysis revealed that NUDCD1 knockdown increased the rate of apoptosis in Patu8988 and PANC-1 cells. (**E**–**N**) Western blot analysis showed that NUDCD1 knockdown decreased the expression of Bcl-2 and increased the expression of Bax and Cleaved Caspase-3 in PANC-1 and Patu8988 cells. **p<0.01, ***p<0.001, ****p<0.0001.

Considering the data from cell proliferation and colony formation assay, we speculated that NUDCD1 could have a role in the apoptosis of PC cells. Thus, we performed flow cytometry to examine the apoptosis of cells transfected with si-NUDCD1. As shown in Figure 3A–3D, NUDCD1 knockdown remarkably increased the rate of apoptosis in PC cells (Patu8988 and PANC-1) relative to the equivalent Si-NC group. Furthermore, we detected the levels of apoptosis-related proteins and found that NUDCD1 knockdown upregulated Bax and downregulated Bcl-2 (Figure 3E–3J).

### NUDCD1 knockdown reduces the invasion and migration abilities of PC cells

**Figure 4:**
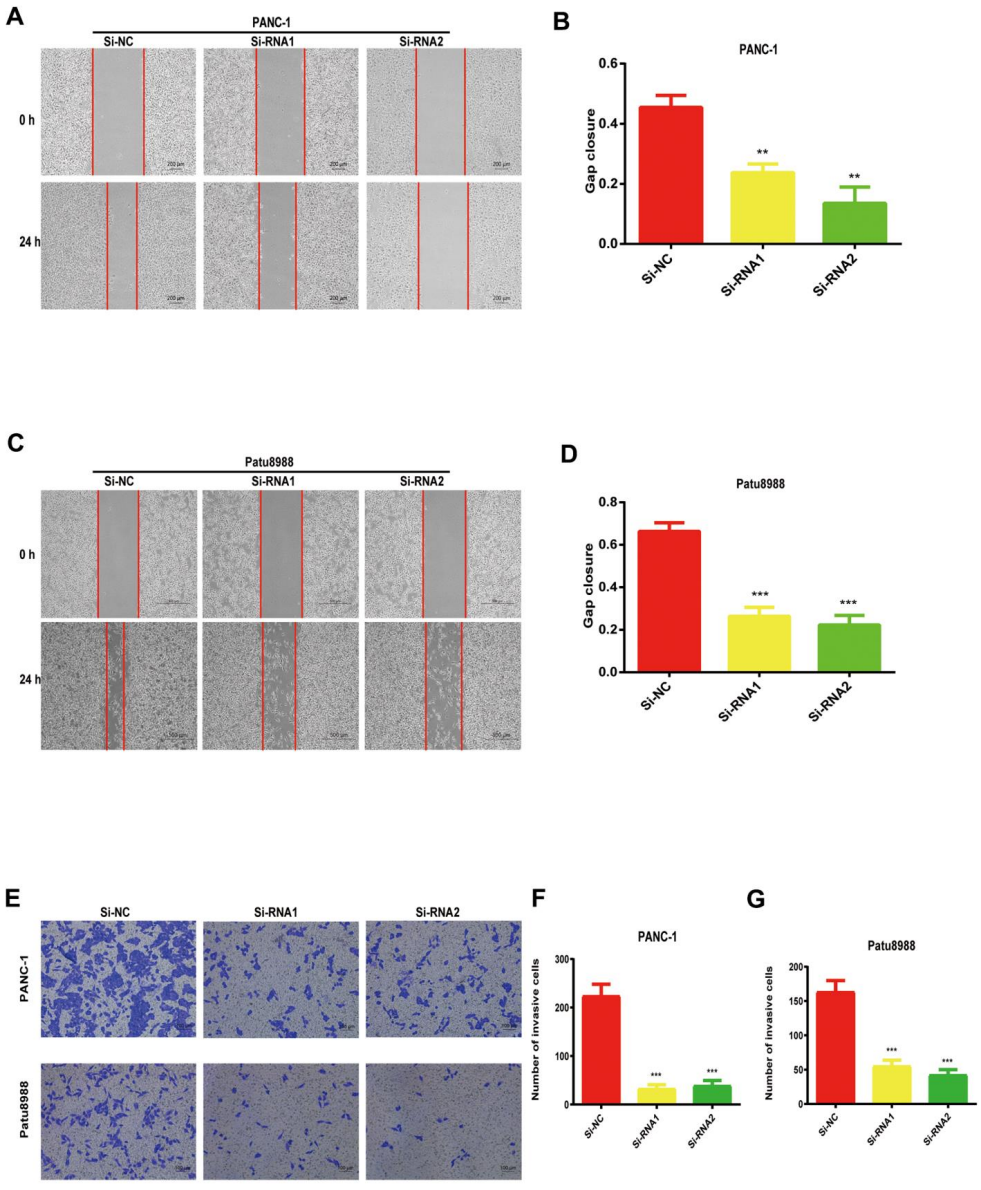
**NUDCD1 knockdown suppressed the invasion and migratory abilities of PC cells.** (**A**–**D**) NUDCD1 knockdown inhibited the migration of Patu8988 and PANC-1 cells in the wound healing assay. (**E**–**G**) NUDCD1 knockdown inhibited the invasion of PANC-1 and Patu8988 cells in the transwell assay. **p<0.01, ***p<0.001.

Considering the data from our colony formation and cell proliferation assays, NUDCD1 may influence the invasion and migration abilities of PC cells. Expectedly, we found that that NUDCD1 knockdown considerably suppressed the migration capacity of Patu8988 and PANC-1 cells relative to the control group (Figure 4A–4D). Similarly, NUDCD1 knockdown overtly inhibited the invasion ability of the cells in comparison to the equivalent Si-NC group (Figure 4E–4G).

### NUDCD1 modulates EMT in PC cells

**Figure 5:**
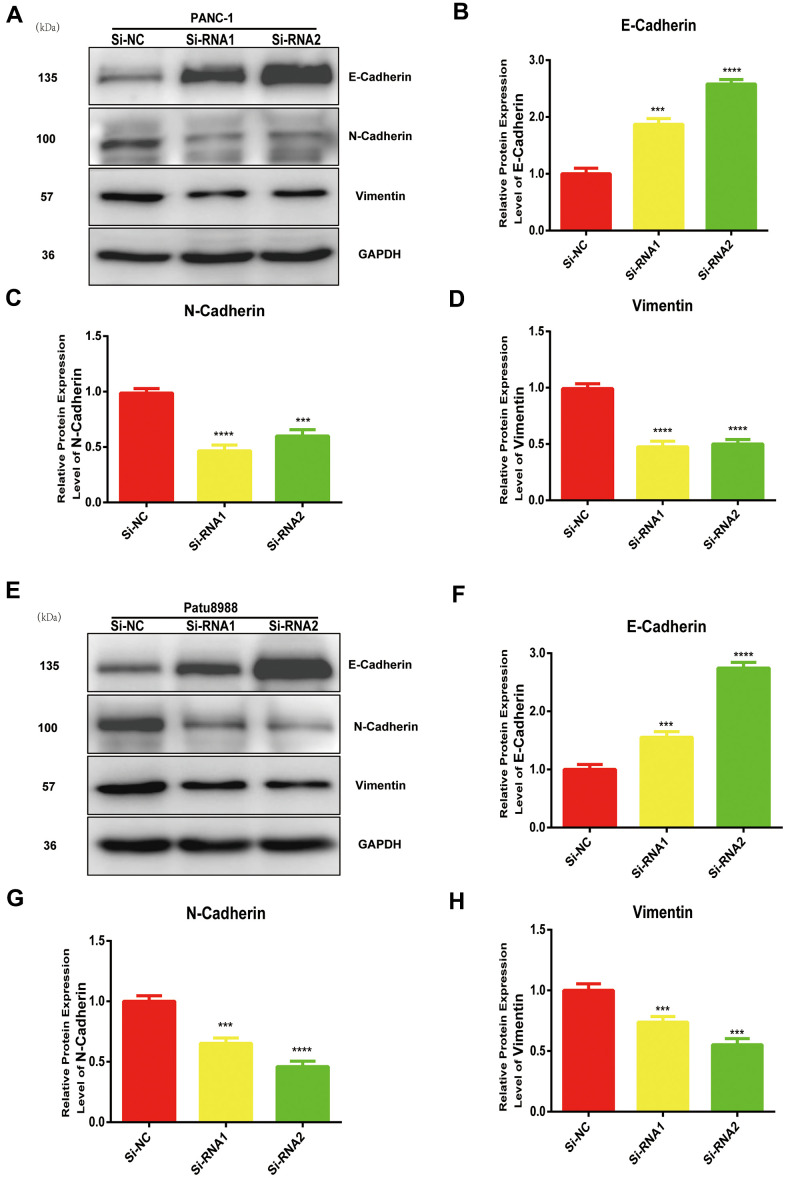
**NUDCD1 knockdown inhibited the EMT process in PC cells.** (**A**–**D**) NUDCD1 knockdown decreased the expression of N-cadherin and vimentin and upregulated the expression of E-cadherin in PANC-1 cells. (**E**–**H**) NUDCD1 knockdown decreased the expression of N-cadherin and vimentin, and upregulated the expression of E-cadherin in Patu8988 cells. ***p<0.001, ****p<0.0001.

EMT is vital in the metastasis of cancer cells. The expression levels of N-cadherin and vimentin were reduced whereas that of E-cadherin increased in NUDCD1 knockdown cell lines (Figure 5A–5H). Collectively, these results indicated that NUDCD1 knockdown may probably inhibit the metastasis of PC cells by modulating EMT.

### NUDCD1 silencing suppresses tumor growth *in vivo*

**Figure 6:**
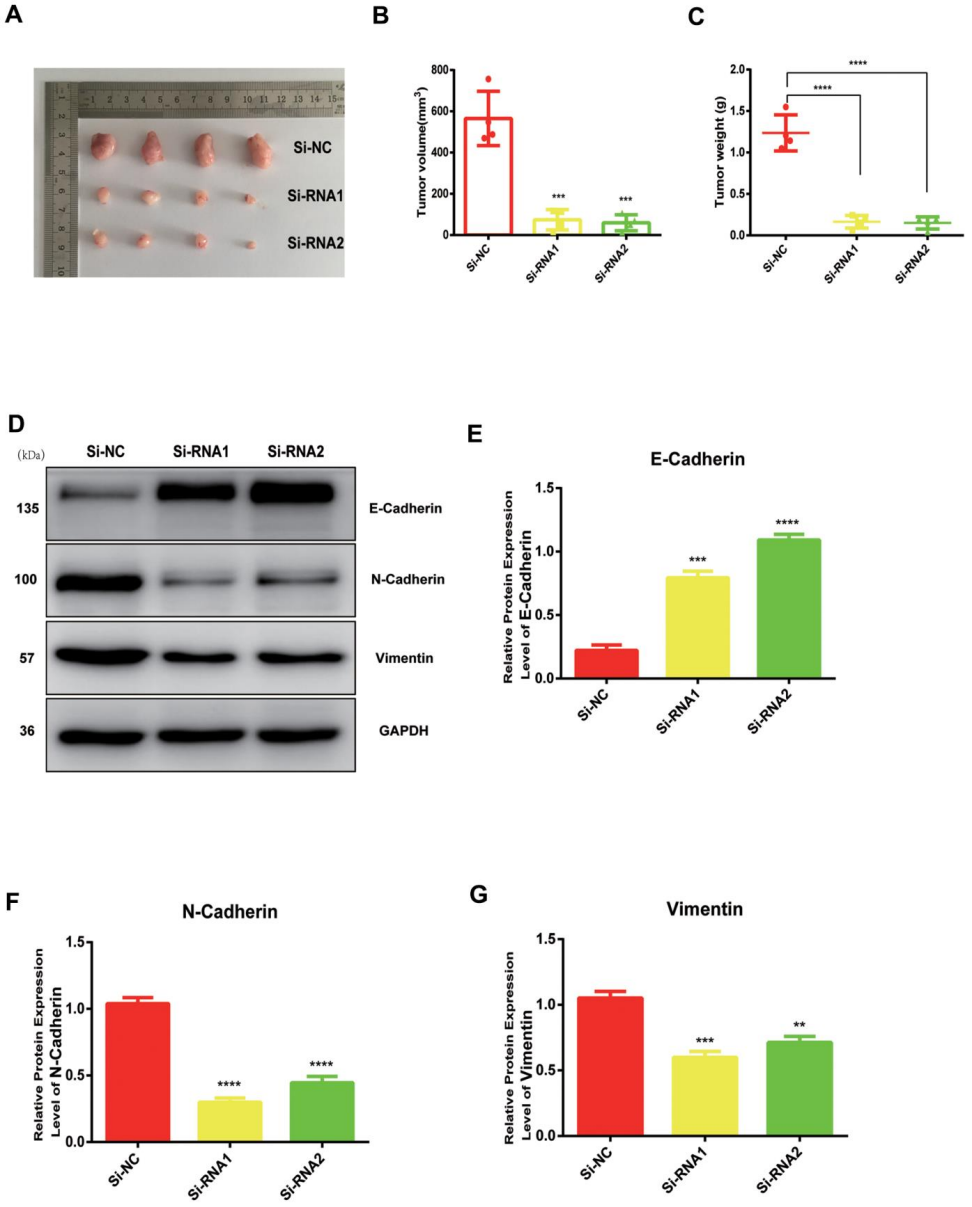
**NUDCD1 knockdown inhibited the growth of PC *in vivo*.** (**A**–**C**) NUDCD1 knockdown remarkably reduced tumor volume and weight. (**D**–**G**) NUDCD1 knockdown decreased the expression of N-cadherin and vimentin and increased the expression of E-cadherin in tumor tissue. **p<0.01, ***p<0.001, ****p<0.0001.

We established a tumor xenograft model ro clarify more clearly the role of NUDCD1 in PC. The examination of subcutaneous tumorigenesis in nude mice revealed that tumor weight and volume were reduced remarkably following NUDCD1 knockdown (Figure 6A–6C). Western blot assay also revealed that NUDCD1 knockdown decreased the protein expression of vimentin and N-cadherin and upregulated that of E-cadherin (Figure 6D–6G). These findings were consistent with those *in vitro*.

## DISCUSSION

PC is one of the quickly growing malignant tumors [12, 13]. The global occurrence rate of pancreatic cancer has increased rapidly in more than 10 years [14, 15]. SLC16A is a common PC biomarker that is used in the classification of PC [16]. NUDCD1 plays a vital function in the pathogenesis of cancer [17, 18]; however, the molecular mechanisms behind its functions in PC remain a mystery. NUDCD1 acts as a crucial component of apoptosis and immune responses, has been identified as an oncoprotein that is frequently activated or upregulated in various human cancers, and is known as an important kind of cancer antigen [18]. In the present study, NUDCD1 was upregulated in PC cell lines and tumor tissues, and its upregulation had a remarkable association with poor prognosis (Figure 1). Moreover, NUDCD1 silencing could inhibit the invasion, colony formation, migration, and proliferative abilities of PC cells (Figures 2, 4).

EMT is considered a major driver of metastasis in PDAC [19, 20]. During invasion and metastasis, epithelial-derived cells lose the characteristics of epithelial cells, reduce intercellular adhesion, express markers of mesenchymal cells and enzymes that decompose extracellular matrix, which eventually leads to increased exercise and invasive abilities [21, 22]. In this study, we found that the downregulation of NUDCD1 could suppress the protein expression of vimentin and N-cadherin and boost the expression levels of E-cadherin (Figure 5). These findings suggested that NUDCD1 could influence PC cell metastasis by regulating EMT. In addition, we conducted animal experiments to explore the role of NUDCD1 in tumorigenesis (Figure 6). As far as we know, the present study is the first to investigate the function of NUDCD1 in PC.

This study was limited because of the small sample size used to arrive at the conclusions. Further studies with larger sample sizes should be conducted to verify our findings.

In summary, the findings of this study showed that NUDCD1 silencing inhibits the invasion, colony formation, migration, and proliferation of PC cells and facilitates cell apoptosis through the EMT pathway. These data provide new insights into the identification of candidate molecular markers for the diagnosis and treatment of PC.

### Ethics statements

The animal study was approved by the Institutional Animal Care and Use Committee of Wenzhou Medical University, China. The methods were performed according to the guidelines approved by the Institutional Review Board of Wenzhou Key Laboratory of Surgery, China.

### Availability of data and materials

The additional data used to arrive at the conclusions can be obtained from the corresponding author upon reasonable request.
